# Increasing Water Temperature Triggers Dominance of Small Freshwater Plankton

**DOI:** 10.1371/journal.pone.0140449

**Published:** 2015-10-13

**Authors:** Serena Rasconi, Andrea Gall, Katharina Winter, Martin J. Kainz

**Affiliations:** WasserCluster–Biological Station Lunz, Inter-university Centre for Aquatic Ecosystem Research, A-3293 Lunz am See, Austria; INRA, FRANCE

## Abstract

Climate change scenarios predict that lake water temperatures will increase up to 4°C and rainfall events will become more intense and frequent by the end of this century. Concurrently, supply of humic substances from terrestrial runoff is expected to increase, resulting in darker watercolor (“brownification”) of aquatic ecosystems. Using a multi-seasonal, low trophic state mesocosm experiment, we investigated how higher water temperature and brownification affect plankton community composition, phenology, and functioning. We tested the hypothesis that higher water temperature (+3°C) and brownification will, a) cause plankton community composition to shift toward small sized phytoplankton and cyanobacteria, and, b) extend the length of the growing season entailing higher phytoplankton production later in the season. We demonstrate that the 3°C increase of water temperature favored the growth of heterotrophic bacteria and small sized autotrophic picophytoplankton cells with significantly higher primary production during warmer fall periods. However, 3X darker water (effect of brownification) caused no significant changes in the plankton community composition or functioning relative to control conditions. Our findings reveal that increased temperature change plankton community structure by favoring smaller sized species proliferation (autotrophic phytoplankton and small size cladocerans), and increase primary productivity and community turnover. Finally, results of this multi-seasonal experiment suggest that warming by 3°C in aquatic ecosystems of low trophic state may cause planktonic food web functioning to become more dominated by fast growing, r-trait species (i.e., small sizes and rapid development).

## Introduction

Global change poses multifaceted threats for aquatic ecosystems. It is expected that water temperatures of lakes can increase by 4°C and precipitation events will change their frequency and intensity during the next century [1]. Over the mid-latitude areas of the Northern Hemisphere, heavy rainfall events have increased steadily since the beginning of the last century and are predicted to get more intense and frequent by the end of this century [2]. Higher precipitation will also lead to higher input of humic substances from terrestrial runoff [3,4], resulting in more colored water, a phenomenon previously termed “brownification” of aquatic ecosystems [5,6], and consequently reduced water transparency and primary production.

Global change can disrupt seasonal successions of phytoplankton community compositions ('plankton phenology'; [7]). Species that grow better at higher temperatures and are better adapted to low-light conditions can gain competitive advantage from such predicted changes in aquatic ecosystems [8], which favor cyanobacteria particularly in shallow lakes [9]. Several studies reported on advanced onsets of phytoplankton blooms as ecological response to global warming and changes in plankton phenology [10–13] with consequent decoupling of trophic interactions at upper trophic levels [14]. Predicted warmer fall and winter periods can induce higher and prolonged phytoplankton productivity [15] and extend the active phase of zooplankton that keep grazing on phytoplankton [10,16–18] as far as nutrients are not limiting. Further to these findings, it is not known yet how darker watercolor as a result of increased precipitation and recharge of drainage water (additional effect of climate change) affects phytoplankton production later in the season.

There is increasing evidence that plankton particle size is affected by climate change induced warming. In general, larger size species are better adapted to colder environments, while species of smaller sizes are more often found in warmer regions [19]. According to the species shift hypothesis, global warming will likely cause decreasing body-size and an overall increase in the proportion of smaller size species at the community scale [20,21] with consequences for community structure and biotic interactions. For example, smaller cells tend to grow faster [22] and are competitively superior to larger cells in their nutrient uptake and growth rate [23,24]. Under higher temperatures, the competitive advantages for nutrient uptake by smaller species are even enhanced [25,26]. Although climate change scenarios include both increases of water temperature and color ('brownification'), it remains still unclear how both these factors affect plankton cell size.

Climate change also affects the composition and activity of aquatic food webs [27,28] with cascading consequences on food web dynamics and element cycling [29,30]. Resulting changes in phytoplankton community structure are expected to affect primary production and push the pelagic food web to shift toward rapid carbon cycling in the microbial loop associated with the dominance of fast growing picoplankton [25,31–33]. In addition, rising temperature stimulates bacterial activity and consequently enhances the flow of organic matter through the microbial loop [34]. Increased precipitation equally results in increased delivery of organic matter from terrestrial sources to lakes ('brownification'), and consequently enhances bacterial biomass [3]. Moreover, higher temperatures favor higher metabolic rates and dietary energy requirements in heterotrophic organisms can exceed dietary supply from autochthonous primary production [25]. Therefore, increasing water temperature and allochthonous organic matter supply can alter the metabolic balance of aquatic ecosystems [35].

Although the effects of extreme weather events are of increasing concern to ecologists [36], the provoked consequences on plankton community dynamics from spring to late fall have not been tested yet empirically. In an effort to better understand mechanisms that drive processes at the planktonic food web level and to test how plankton respond to physico-chemical changes, mesocosm experiments have been widely used and their results certainly advanced our knowledge of aquatic food web processes [37–40]. Encouraged by such recent advances, we designed a multi-seasonal (8 months) mesocosm experiment to test how increasing water temperature and brownification affect plankton community composition, phenology, and functioning. Recognizing that current studies on effects of brownification were conducted in already nutrient-rich and/or colored aquatic ecosystems [4,41], we asked how clear water systems respond to increasing watercolor and temperature. We thus hypothesized that increasing water temperature and brownification will, a) cause plankton community composition to shift toward small size phytoplankton and cyanobacteria [9], and, b) extend the length of the growing season entailing higher phytoplankton production later in the season.

## Methods

### Experimental setup

Twenty-four thermally insulated cylindrical polyethylene containers (74 cm diameter x 102 cm height) were placed outside the research center WasserCluster Lunz (47°51′ N, 15°01′ E) and each filled with and kept at 400 L of surface lake water from nearby Lake Lunz. Lake zooplankton was collected using a zooplankton net (100 μm mesh size), pooled in a bucket, and subsequently equally distributed to each of the mesocosms. Collecting permits were provided through an agreement between the owner of Lake Lunz and WasserCluster Lunz. None of the species collected are considered threatened. Treatments (6 replicates) consisted of controls (C, ambient watercolor and temperature), elevated temperature (T, +3°C above control temperatures), brownification (B, 3x higher water color relative to control) and a treatment with both elevated temperature and watercolor (TB). The experiment lasted from April 18 to December 12, 2013.

Each of the mesocosms was permanently and equally aerated by air diffusers and protected from external input of particles by a nylon mesh at the top. As in other studies, the enclosure walls were regularly removed to minimize the growth of periphytic algae (see [42]), which settled to the bottom. Nutrient (P and N) concentrations were measured weekly and all mesocosms were equally fertilized according to the Redfield ratio (3 μg P from K_2_PO_4_ and 45 μg N from NaNO_3_) to avoid nutrient depletion throughout this experiment. In contrast to most experiments that tested effects of climate change on plankton in nutrient-rich systems [43–45], our study was conducted in systems maintained at mesotrophic conditions, as is the case in many shallow lakes and ponds in mostly pristine aquatic ecosystems [46].

Water temperature of all mesocosms was controlled by a computerized system as described elsewhere [42]. Watercolor was measured every week spectrophotometrically (420 nm wavelength) and the brownification treatment was maintained by adding weekly a humic acid solution (Humin feed–HuminTech, Germany) to maintain a 3X higher watercolor compared to the control.

### Samples analysis

Samples were taken fortnightly from each mesocosm using a plastic tube (100 cm length, 6 cm diameter, ~3 L volume) and processed for analyses the same day. NO_2_-N, NO_3_-N and NH_4_-N were analysed using a continuous flow analyzer (FlowSys, Systea). Total phosphorus (TP) was quantified after persulfate digestion followed by molybdate reaction (Wetzel and Likens 2003) and soluble reactive phosphorus (SRP) after filtration of acid washed GF/F filters and molybdate reaction (Wetzel and Likens 2003) at 880 nm wavelength using a UV/VIS spectrophotometer (UV-1700). Dissolved organic carbon (DOC) was measured after filtration on pre-combusted GF/F filters using a TOC analyzer (Sievers 900, GE).

Zooplankton were collected by sieving 10 L of water through a 100μm mesh and taxonomically identified using a stereo microscope (Bresser microscope, Germany) at 40x magnification. Subsequently, phytoplankton (<100 μm) were fixed with Lugol, and variable volume (5 to 50 μL) was settled following the Utermöhl method [47]. Samples were counted on an inverted microscope (Leica DMI 3000 B) and at least 400 cells were identified to the genus level. Phytoplankton biovolumes were assigned using reference data [48]. Heterotrophic and autotrophic plankton were fixed with formaldehyde and counted in triplicates using a flow cytometer (Cell Lab Quanta, Beckmann Coulter). These picoplankton were separated based on differences in fluorescence and the cytometric raw data subsequently analyzed using the software Cell Lab Quanta SC.

As a proxy for phytoplankton diversity, we used the genus richness (number of genera) and evenness (ɛ), calculated using the biovolume repartition among genera of cell dimensions 5–100 μm. For zooplankton, we used the counts of individuals identified at the genus level. Phytoplankton size distribution was separated into three different size classes: pico (0–5 μm), small (5–20 μm) and large (20–100 μm) plankton size.

Phytoplankton productivity was assessed using a Phyto PAM analyzer system (Walz, Germany) and primary production was estimated using the formula indicated elsewhere [49]. The cell-specific photosynthetic efficiency (mg C h^-1^L^-1^ (μg C cells^-1^)) was calculated as the quotient of estimated primary production and phytoplankton biomass [50].

Species turnover was calculated as Bray Curtis community dissimilarity between subsequent sampling dates and averaged over the different seasons of the experiment.

### Statistical analysis

Data were analyzed using R software (http://www.r-project.org), including the packages “DoBy” for data formatting, “Vegan” for multivariate statistics and “mgcv” for generalized additive models (GAM) computation and visualization. To test for the effects of increased watercolor and warming over this investigated time period (months), we used Wilcoxon test (non-parametric statistical hypothesis test used for comparing repeated measurements). We fit GAM models using measured data to assess the nonlinear relationships between the smoothed abundances of plankton communities (bacteria, phytoplankton, zooplankton) and temperature and watercolor as the environmental forcing stressors. The dataset was analyzed entirely for temporal patterns and dynamics, while testing for treatment effects we grouped the different seasons of the experiment (spring, summer, and fall). The fall months of the experiment (from September 23 to December 12) showed the most significant effect and were thus particularly used for comparisons among treatments. All data were analyzed untransformed.

## Results

### Physico-chemical parameters

**Fig 1:**
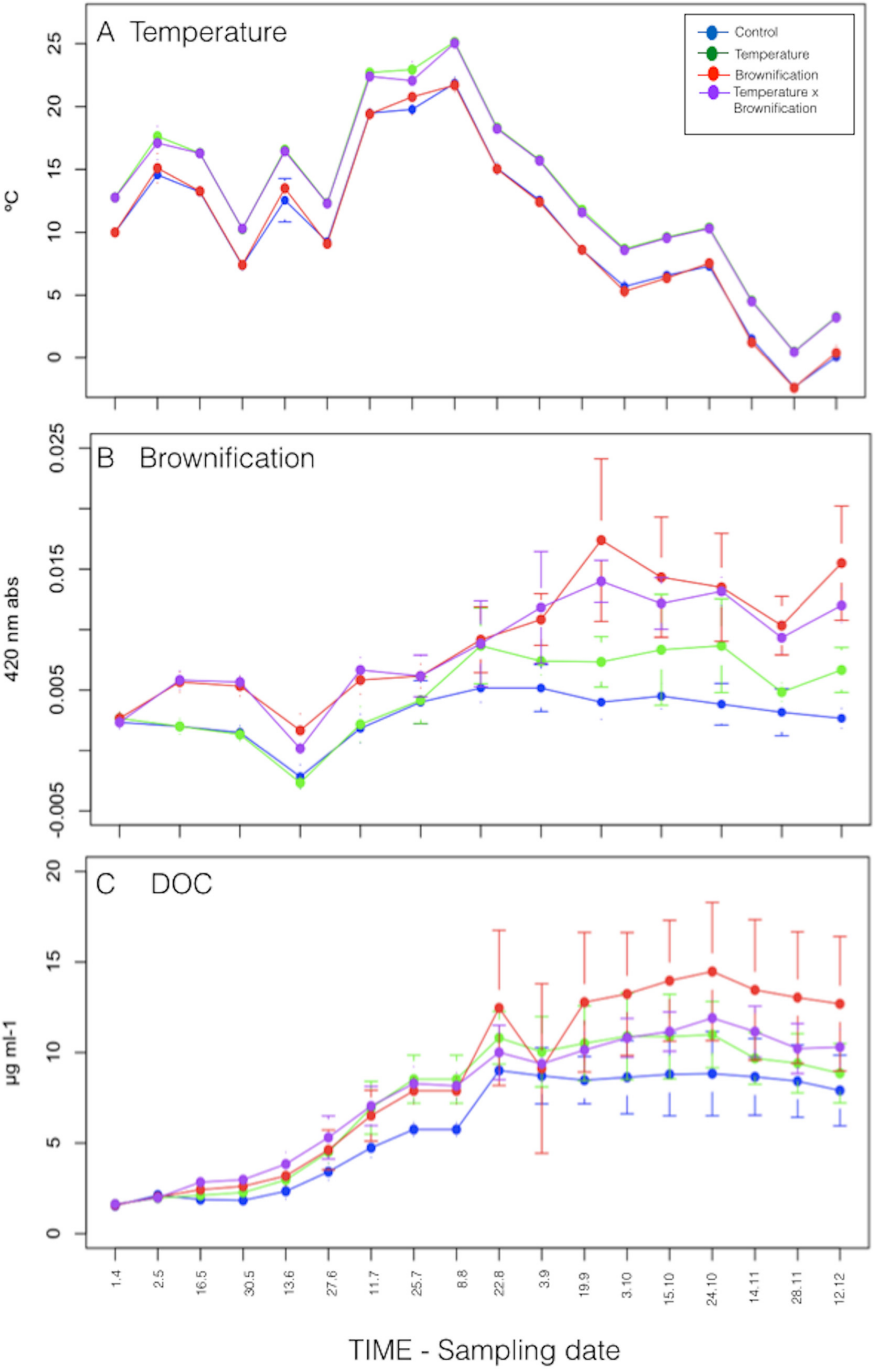
Physico-chemical parameters. Temporal dynamic of temperature (a), watercolor (b) and dissolved organic carbon (DOC)(c).

Water temperatures were continuously kept +3°C higher in the heated treatments (T and TB) than in C and B (Fig 1A). Watercolor (measured as light absorbance at 420 nm) was kept constantly ~3X higher in the brownified treatments (B and TB) than in C and T (Fig 1B). Dissolved organic carbon (DOC) concentrations increased from the end of July (Fig 1C), coinciding with the onset of phytoplankton growth, and remained higher in all treatments compared to C during the entire experiment. However, there was no significant difference in dissolved organic carbon (DOC) concentrations among the treatments during each sampling throughout the 8 months experiment (1.5–14.5 μg L^-1^).

Concentrations of soluble reactive phosphorus (SRP) ranged from 0.02–75.5 μg L^-1^ and total phosphorus (TP) from 0.17–186 μg L^-1^, and covered thus a wide seasonal span from oligo- to meso/eutrophic status. Average SRP concentrations were significantly lower in T compared to C (P<0.001; lm-ANOVA), while TP concentrations were significantly higher (P<0.001; lm-ANOVA). Nitrite and ammonium concentrations were similar (0.1–67.8 μg L^-1^ and 1.1–93 μg L^-1^, respectively) and generally lower than nitrate concentrations (0.1–2302 μg L^-1^; S1 Table). Nitrite concentrations were significantly lower in T, B, and TB than in C (P≤0.001; lm-ANOVA), while nitrate concentrations were significantly higher in T, B, and TB than in C (S1 Table).

### Plankton phenology

**Fig 2:**
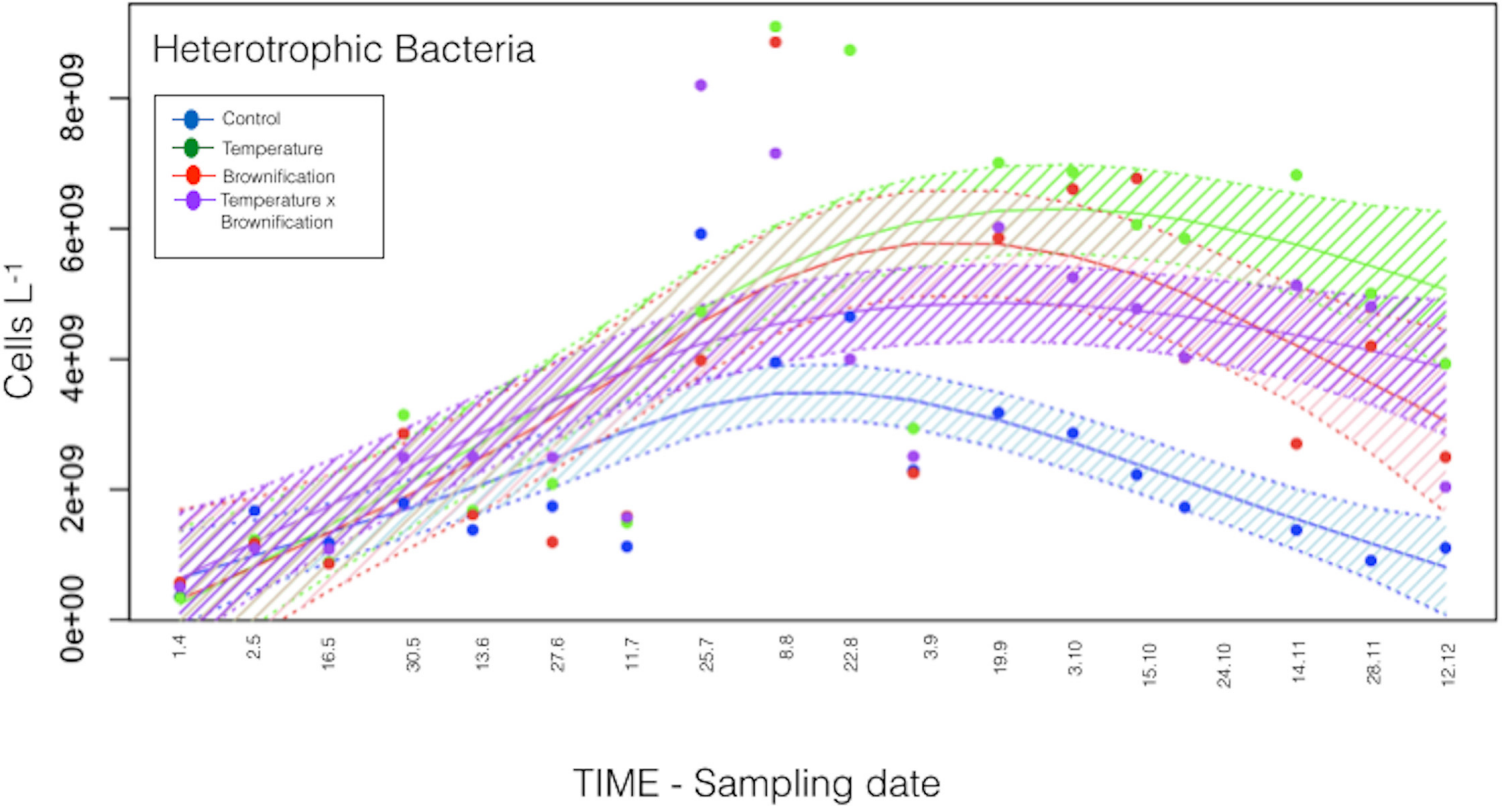
Plankton phenology. Generalized Additive Model for heterotrophic bacteria. The shaded area indicates 95% of confidence interval.

Heterotrophic picoplankton (bacteria) abundance (Fig 2) increased from initially 3.29 × 10^8^ cells L^-1^ to 5.56 × 10^9^ cells L^-1^ by mid July. As of August, average bacteria abundance was higher in T, B, and TB (5.49, 4.76, and 4.13 x 10^9^ cells L^-1^, respectively) compared to C (2.20 x 10^9^ cells L^-1^). The highest abundance was generally recorded in T and, during fall, significantly higher than in C (P<0.001; Wilcox test). Bacteria abundance was also significantly higher in B and TB (P<0.001) compared to C.

**Fig 3:**
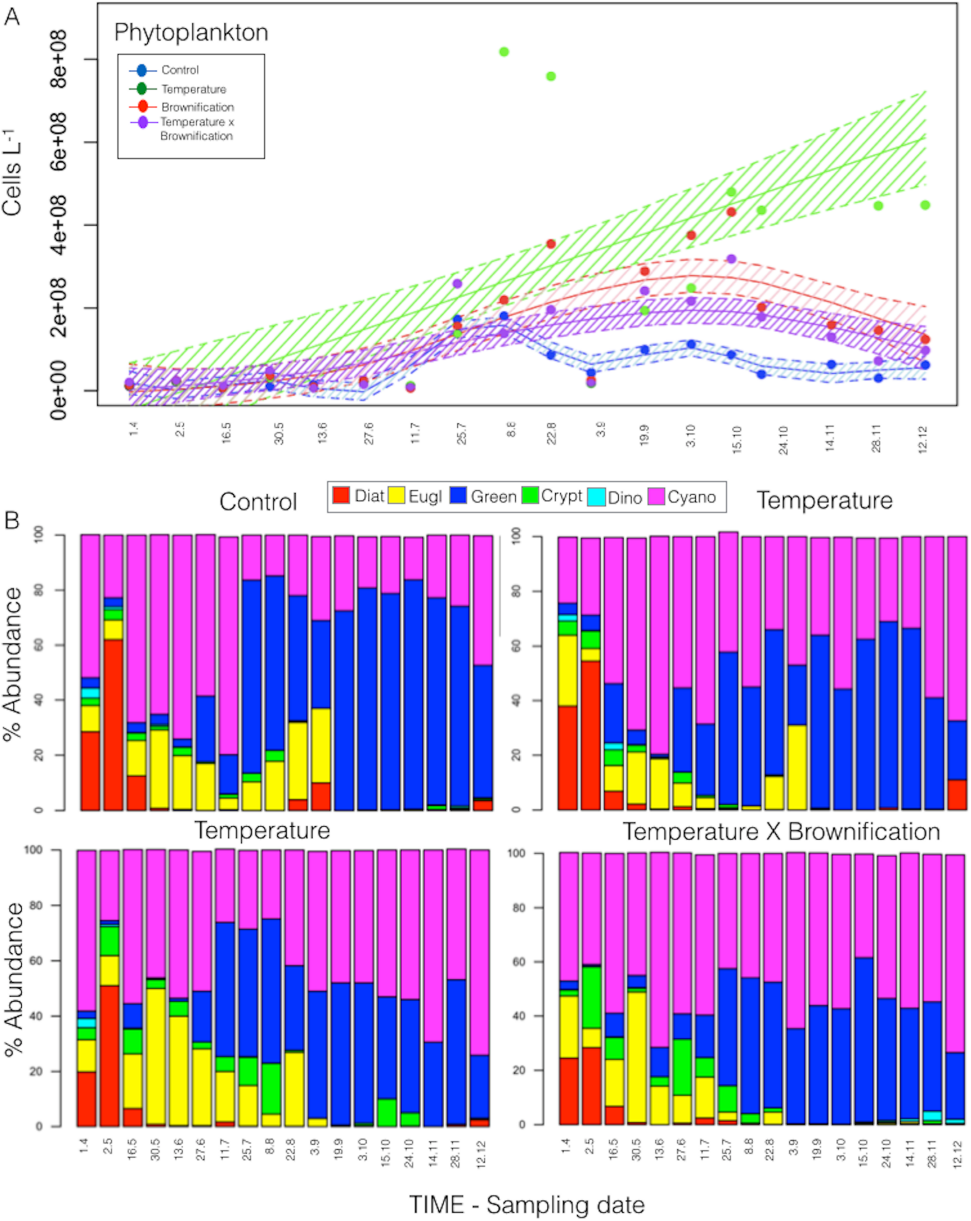
Plankton phenology. Generalized Additive Model for phytoplankton abundance (a) and relative taxa composition (b). The shaded area indicates 95% of confidence interval.

Phytoplankton abundance, including picoautotrophs (Fig 3A), spanned over two orders of magnitude and ranged from 5.9 × 10^6^ cells L^-1^ (C in May) to 9.58 × 10^8^ cells L^-1^ (T in late October); as of mid July, the phytoplankton abundance was ~4X higher in T, B, and TB (29.12 ± 2.01 x 10^7^ cells L^-1^) than in C (8.03 ± 4.41 x 10^7^ cells L^-1^). Elevated temperature had the strongest effect on increasing phytoplankton abundance (P<0.001; Wilcox test), based on significant differences compared to C; thus, phytoplankton abundance in T remained high during most of the experiment.

Phytoplankton abundance in C peaked in early August (mainly green algae, picophytoplankton, and traces of diatoms) and October (mainly gelatinous green algae and cyanobacteria, together with picophytoplankton). Similarly, in the temperature treatments, phytoplankton abundance was highest in August with mainly picophytoplankton as well as colonial and filamentous cyanobacteria, including *Aphanotece* and *Cylindrospermum*, and in November with high abundance of small green algae (*Selenastrum* and *Scenedesmu*s) and picophytoplankton (<5 μm) (Fig 3B). We also observed the growth of periphytic algae (*Mougeotia sp*.) on the enclosure wall from May to October, but no significant difference was detected in periphyton growth among the treatments (P>0.3, Wilcox test).

**Fig 4:**
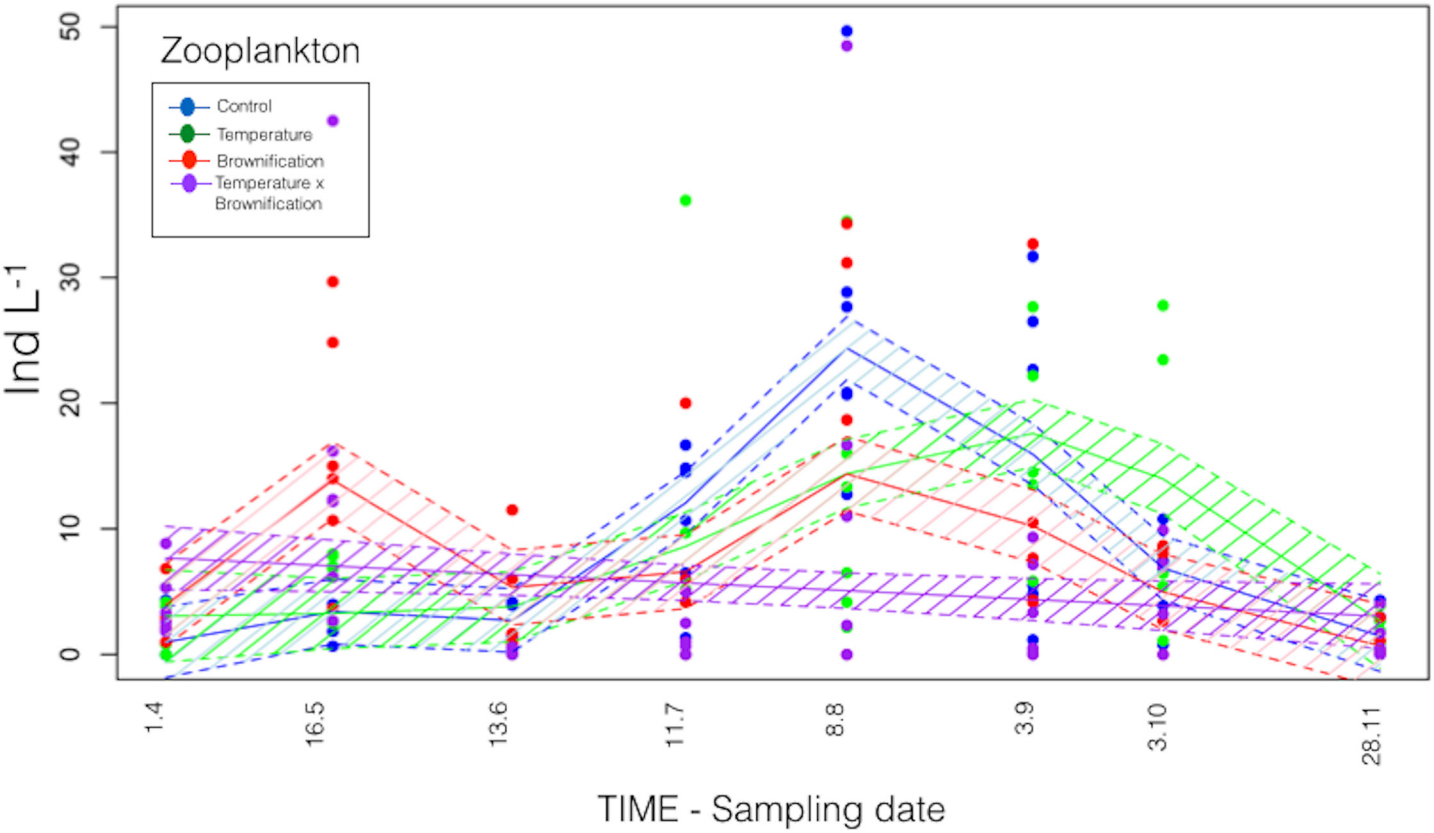
Plankton phenology. Generalized Additive Model for zooplankton abundance dynamics over time. The shaded area indicates 95% of confidence interval.

Zooplankton abundance ranged from <1 ind. L^-1^ in July to 27 ind. L^-1^ in September (both in C) (overall mean 7.5 ± 6.9 x 10^8^ ind. L^-1^). In general, zooplankton abundance was lower at the beginning of the experiment, except for a peak in May in B and TB (Fig 4), with predominantly *Daphnia longispina* and *Bosmina longirostris*. In October, higher zooplankton abundance was recorded in T and TB, but not significantly different from C (P>0.05; Wilcox test). In general, higher abundance of small cladocerans (mostly *Bosmina*; Ø = 0.5 mm body length) was detected in T.

Among all treatments, heterotrophic bacteria abundance were significantly explained (P≤0.01; GAM models) by temperature (44.6% of variance explained) and DOC concentrations (32.3%, P≤0.01; GAM models), whereas phytoplankton abundance, tested separately from the physico-chemical parameters, explained 79.1% of variance in heterotrophic bacteria biomass (P≤0.0001; GAM models). Changes in temperature and total phosphorous concentrations (TP) significantly explained changes in phytoplankton abundance (42.6% and 39.5% of the variance, respectively; P≤0.01; GAM models). Among other planktonic compartments, heterotrophic bacteria explained 62.1% of the variance in phytoplankton dynamics (P≤0.0001; GAM models). However, zooplankton abundance was not significantly explained by changes in temperature, watercolor, or phosphorus (P>0.1; GAM models), but only total phytoplankton abundance significantly (P<0.001) explained the variance in zooplankton abundance (61.2%) (Table 1).

**Table 1:** Table of results for significant factors involved as a driver of temporal changes for the studied planktonic compartments.

	% Deviance explained	p-value	Significance code
**Heterotrophic bacteria abundance *vs***			
Temperature	44.6	0.008	**
DOC	32.3	0.006	**
Phytoplankton abundance	79.1	<0.001	***
**Phytoplankton abundance *vs***			
Temperature	42.6	0.01	*
TP	39.5	0.006	**
Heterotrophic bacteria abundance	62.1	<0.001	***
**Zooplankton abundance *vs***			
Heterotrophic bacteria abundance	61.2	0.001	**

Significance codes

***p< 0.001

** p<0.01

* p<0.05.

### Diversity indices and community structure

**Fig 5:**
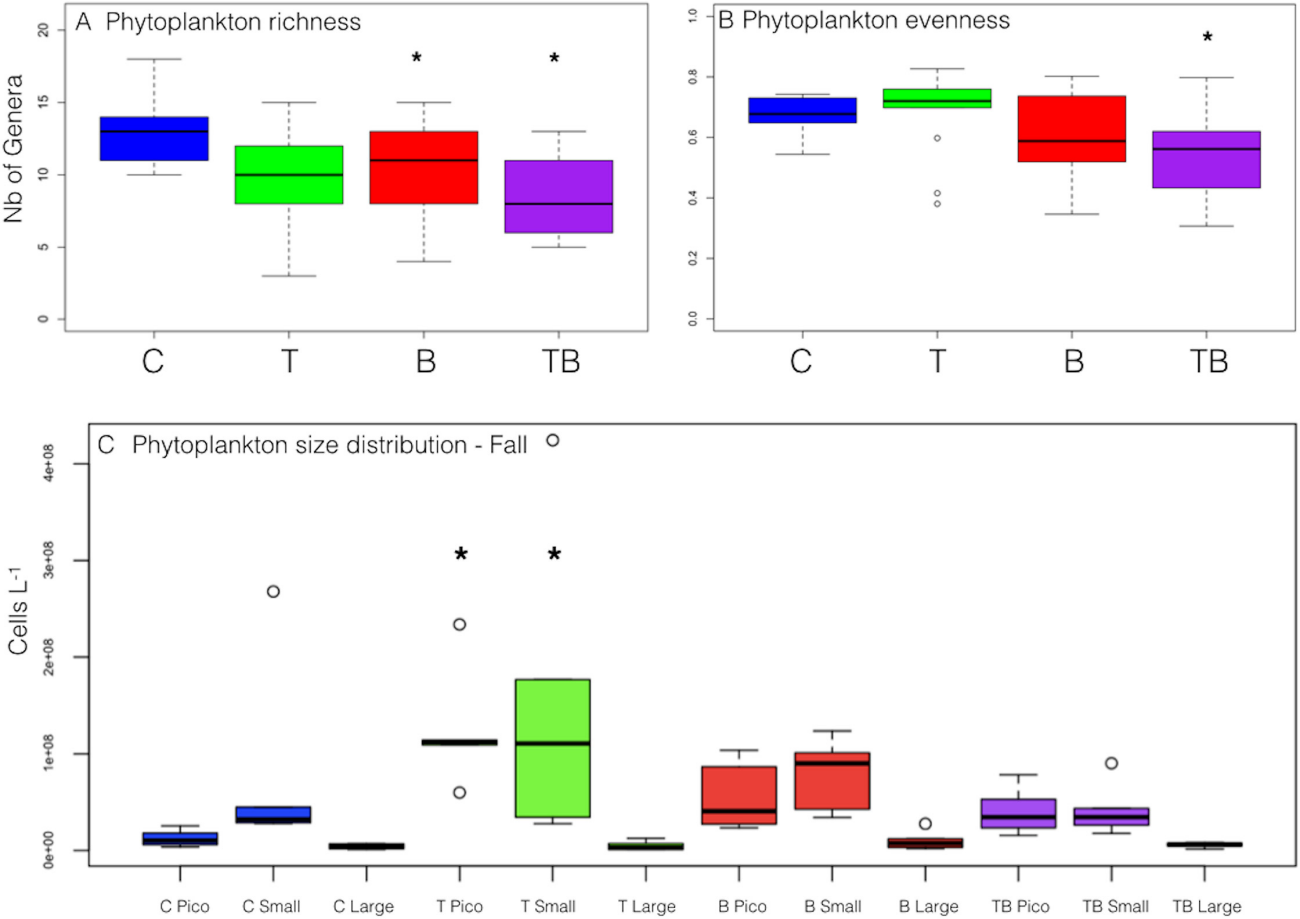
Phytoplankton community structure. Boxplots representing phytoplankton richness (a), evenness (b), and phytoplankton biovolume size distribution (c) at the end of the experiment. Pico = 0–5 μm, Small = 5–20 μm, Large = 20–150 μm average diameter.

Phytoplankton diversity (Shannon index) declined continuously from spring to late fall and significantly among all treatments (P<0.01, Wilcox test). Particularly during fall, richness was highest in C and decreased in all treatments. Compared to C, the genus richness was significantly lower in T (P<0.01; Wilcox test) and TB (P<0.004; Wilcox test) (Fig 5A), mainly due to the loss of diversity among diatoms and Chlorophyta. Phytoplankton evenness (Fig 5B) was significantly lower only in the TB treatment (P<0.005; Wilcox test).

There was a clear effect of the higher temperature on phytoplankton size structure that resulted in a significantly higher abundance of smaller species (i.e., picoautotrophs and nanophytoplankton; P<0.002; Wilcox test) than in the ambient temperature treatments. The abundance of picoautotrophs was also significantly lower in C than in B (P<0.004; Wilcox test) and TB, although the latter was not significantly different (Fig 5C).

### Primary production

**Fig 6:**
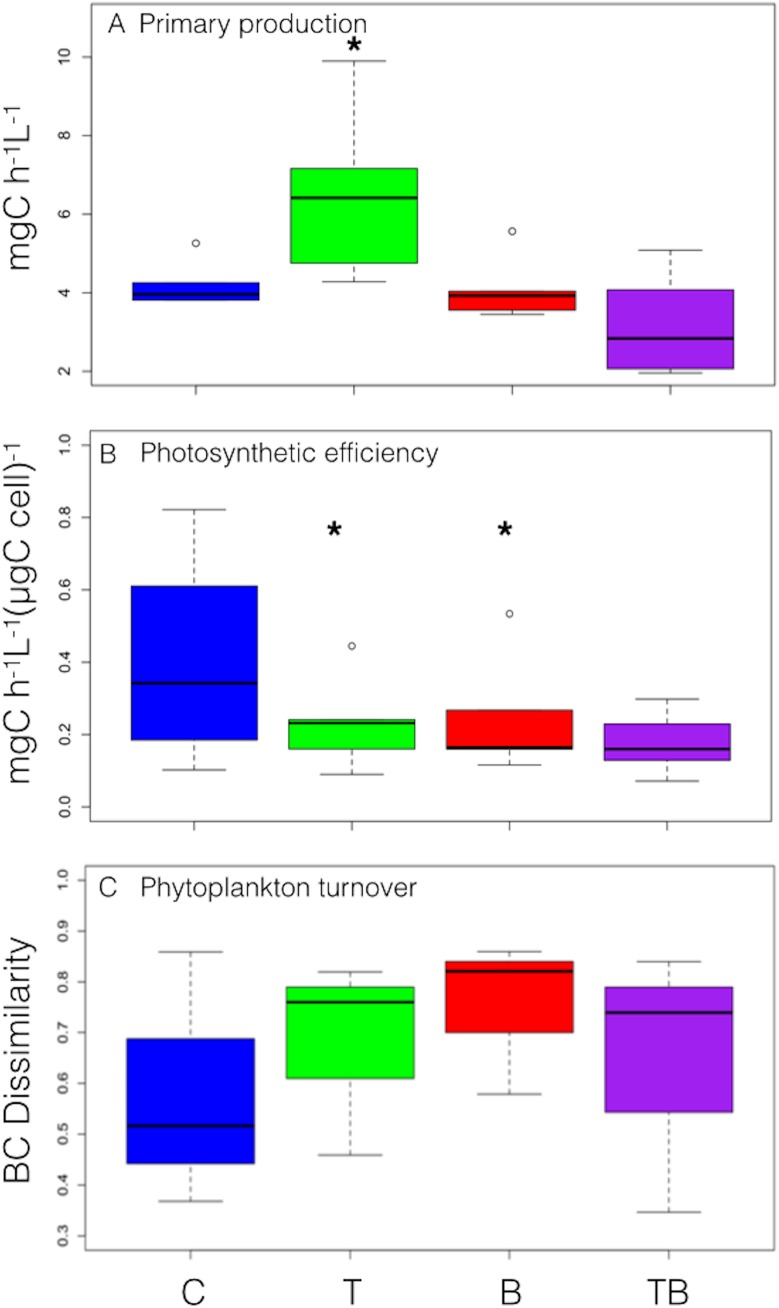
Phytoplankton functional parameters. Boxplots representing the maximum estimated primary production (a), photosynthetic efficiency (b), and phytoplankton community turnover (c).

Estimated phytoplankton primary production decreased after May and remained mostly <5 mg C h^-1^ L^-1^ in all treatments. During fall, the estimated phytoplankton primary production in C was significantly higher in T (P<0.05, Wilcox test), similar in B and lowest in TB (Fig 6A). The photosynthetic efficiency during fall was highest in C, lower in T and significantly lower in B and TB (P<0.01 Wilcox test; Fig 6B). At elevated temperature, higher production was associated with low efficiency, while the combined treatment (TB) resulted in lower production associated with lower photosynthetic efficiency (Fig 6B). The elevated temperature effect on the primary production was associated with high community turnover throughout the entire experiment (Fig 6C).

## Discussion

As hypothesized, this multi-season study demonstrates that increased water temperature favored heterotrophic bacteria and phytoplankton abundance and primary production during fall. We show that higher phytoplankton abundance was associated with higher community turnover, but decreased phytoplankton diversity and photosynthetic efficiency. Moreover, this study provides experimental evidence that increased fall temperatures triggers dominance of significantly smaller phytoplankton size species, whereas brownification of water (3X darker watercolor) did not cause significant differences in heterotrophic bacteria or phytoplankton abundance relative to the control.

### Plankton phenology

The highest effect in stimulating planktonic population growth was due to increased temperature (+3°C), while 3X darker watercolor had only a significant effect on heterotrophic bacteria growth. These results suggest that the effect of brownification on such clear water, oligo-mesotrophic pre-alpine ecosystems, is different to many eutrophic shallow lakes [[43–45]] and northern latitude freshwater systems [46]. As also confirmed by the lack of significant changes in DOC, we suggest that the small amount of humic substances, that pre-alpine aquatic systems typically receive from their generally steep and shallow soil drainage basins, is too small to affect light and nutrients induced changes in plankton phenology.

Higher temperatures in T likely favored higher prokaryotic metabolism [35] and thus higher heterotrophic bacteria abundances. In addition to the direct effect of increased temperature, increased heterotrophic bacteria abundance was associated with increased phytoplankton abundance (lm, P<0.001), so higher heterotrophic bacteria abundance was most likely linked to increased phytoplankton biomass and its enhanced nutrient and readily available DOC release [51].

The phytoplankton abundance in C followed a typical seasonal dynamic of oligotrophic systems [52], and included some species that are characteristic of nearby Lake Lunz (e.g., *Oocystis* and *Aphanoteche*, unpubl. data), and thus shows that these results are similar to oligotrophic, pre-alpine lakes. The lack of temperature-induced earlier phytoplankton spring bloom shows that earlier spring warming without simultaneous increase of nutrients does not cause any earlier phytoplankton bloom in oligotrophic ecosystems [53]. In addition, faster phosphorus uptake (SRP; S1 Table) at higher temperatures suggests that such increased temperature promoted fast growing species that are more competitive in nutrient uptake, such as the small green algae *Selenastrum*, *Scenedesmus* and representative of *Chlorococcales ssp*. The continuous phytoplankton abundance increase towards the end of the experiment confirmed our hypothesis and clearly suggests that warmer water supports phytoplankton growth later in the season and probably provoke a shift of the community composition towards species able to bloom in very short time period, even during winter time. In B, however, the observed phytoplankton peak in October was higher than in TB, indicating a typical fall community composed of species better adapted to a shorter photoperiod and lower temperatures (as *Rhodomonas ssp*. and big desmids).

Zooplankton abundance in C was strongly associated with phytoplankton biomass (mostly diatoms and green algae), whereas in all the other treatments zooplankton (mostly cladocerans) phenology was mainly linked to heterotrophic bacteria abundance. In T, the high zooplankton abundance in October coincided with the maximum heterotrophic bacteria abundance that likely supported the cladocerans growth, as warmer temperatures at this time coincided with colonial and filamentous cyanobacteria that were not exploited by grazers. Similarly, in B zooplankton were likely supported by the higher heterotrophic bacteria abundance. Heterotrophic bacteria are more efficient than phytoplankton in nutrient uptake [22] and the increased amount of humic substances was enough to promote heterotrophic bacteria growth to support zooplankton. However, in this study we did not count other heterotrophic microbial eukaryotes, such as ciliates or other bacterivores, which could also be influenced by warmer temperature in their growth and grazing rate [54]. It is thus possible that zooplankton were not feeding directly on heterotrophic bacteria that do not increase zooplankton survival [55], but perhaps indirectly via bacterivorous protists that support trophic energy transfer [sensu Bec et al [56] and lead to enhanced secondary production.

### Phytoplankton diversity

Phytoplankton diversity indices (i.e. genera richness) decreased in T, B, and TB, but not in C. This higher diversity in C was mostly due to diversified diatoms and dinoflagellates, which are very sensitive to increased temperature and water color [57]. Chlorophyta and Cyanobacteria were the most diversified groups in T and B, respectively, and similarly with reports from marine [58,59] and terrestrial ecosystems [60], in which increasing temperature also increased species richness.

In lakes, progressive loss of phytoplankton diversity is often linked to a shift towards the dominance of cyanobacteria [9], which usually proliferate in warmer waters [8]. In our experiment, cyanobacteria were part of the phytoplankton summer bloom in T, confirming recent theories that higher water temperatures boost cyanobacteria dominance in shallow lakes [9]. However, we did not observe any single species to dominate or bloom. Long-term studies on Lake Müggelsee, Germany, with an average lake depth of 5 m, suggested that increased temperatures were not directly promoting the dominance of cyanobacteria, whereas total phosphorous and water column stratification were the most important triggers [61,62]. Our results provide evidence that in shallow oligotrophic ecosystems, as tested by our mesocosms, higher temperature affects phytoplankton diversity by shifting phytoplankton community towards an increase of picoautotrophs. In the increased watercolor treatments, the significant decrease of species number suggests that decreased light availability, especially during the already shorter photoperiod in fall, further selected for the species that are able to compete for light. However, the remaining species among all treatments were similarly distributed, which indicates that increasing temperature or watercolor did not affect the equal repartition of the species.

In accordance with our hypothesis, we observed the plankton size distribution to shift toward smaller size species at elevated temperatures. There is increasing evidence that a major effect of temperature on plankton community entails changes in community size structure and that global warming benefits the abundance of small-sized species [20,21]. The shift that we observed in T was due to increased picophytoplankton (<5μm) biomass, together with small green algae, such as *Selenastrum sp*. and *Spaherocystis sp*., compared to large diatoms in C. Moreover, an increase in the proportion of smaller-sized species was detected in the plankton community at both the producer and consumer levels and the shift to smaller-sized phytoplankton species in T was associated to smaller-sized zooplankton, mostly *Bosmina*, while larger *Daphnia* were more abundant in C and B. Such smaller plankton size species growth may be attributable to the higher metabolic rates at elevated temperatures that result in higher abundance, but smaller plankton cell size [63]. The higher abundance of smaller-sized herbivorous zooplankton at elevated temperature can also be a consequence of lower food quality associated to small-sized phytoplankton [15].

### Primary production and community turnover

Higher primary production during the +3°C warmer fall may have been directly linked to increased photosynthetic carbon assimilation at higher temperature [64] and higher metabolic rates [25]. Moreover, the higher phytoplankton productivity in T during fall may also be a consequence of different phytoplankton community composition at elevated temperatures. The size structure community shift in T was due to a plankton community dominated by smaller species, better adapted to higher temperatures and may have thus provoked a shift in the functioning of planktonic food webs more dominated by microbial processes. Such proposed shift is indeed possible because the increased primary production was not associated with an increase in the photosynthetic efficiency, but with higher community turnover, typical of communities composed of small cells that tend to grow faster and are competitively superior to larger cells in nutrient uptake and growth rate [22,23].

Our results suggest that +3°C higher temperatures pushed the planktonic food web to shift toward a more rapid carbon cycling associated with the dominance of fast growing species. Therefore, organic matter flow was strongly driven through the microbial loop, resulting in a fast growing, but less performing community in terms of using the available resources to sustain higher trophic levels [25,32,34]. We speculate that the increased primary production did not lead to an increased carrying capacity of these systems. The changes induced by higher temperatures could reflect an alteration of the metabolic strategy at the community level [35], with most of the energy invested to obtain the highest possible yield rather than complex biomass structure [65].

## Conclusions

This multi-seasonal experiment shows that increasing water temperature changes the size structure and functioning of planktonic communities. The strongest effect on the investigated functional properties was detected during late summer and fall, where warming favored the growth of smaller sized heterotrophic bacteria and autotrophic picophytoplankton. This size shift was associated with lower phytoplankton community diversity, but higher primary production, which was related to higher community turnover and fast growth rates rather than to increasing photosynthetic efficiency. These results show linkages between increased temperature and changes in plankton structure (size distribution) and function (productivity and turnover) that are essential to support food web functioning, maturation and stability. Considering that the detected changes in the functional parameters in the temperature treatment are consistent with theories on ecosystem development ([62]; Table 2), we conclude that warming of aquatic ecosystems by 3°C may shift to a planktonic food web functioning more dominated by fast growing, r-trait species (i.e., small sizes and rapid development). Finally, further experimental studies, at perhaps larger scales, are strongly encouraged to better understand and predict effects of climate change on food web interactions at higher trophic levels in oligotrophic freshwater ecosystems.

**Table 2:** Summary of effects of the different treatments on the functional properties of the ecosystem investigated in this study. C = control; T = temperature (+3C); B = brownification; TB = temperature x brownification treatment.

	Species size	Shannon diversity	Production	Photosynthetic Efficiency	Species turnover
C	+	+	-	+	-
T	-	-	+	-	+
B	-	+	=	-	+
TB	-	-	-	-	+

## Supporting Information

S1 Table Raw physico-chemical data from all mesocosms sampling and standard deviations. C = control; T = temperature; B = brownification; TB = temperature x brownification treatment.
(XLSX)
